# A Pore or not a Pore? Understanding Pore Size Distributions of Non‐Graphitic Carbon and Atomically‐Dispersed M‐N‐C Materials

**DOI:** 10.1002/advs.76048

**Published:** 2026-06-12

**Authors:** Simon W. J. Dietzmann, Asad Mehmood, Jian Liang Low, Shu‐Han Wu, Carsten Prinz, Ana Guilherme Buzanich, Jörg Radnik, Paul A. Appel, Franziska Emmerling, Tim‐Patrick Fellinger

**Affiliations:** ^1^ Division For Electrochemical Energy Materials Bundesanstalt für Materialforschung und ‐prüfung (BAM) Berlin Germany; ^2^ Department of Chemistry Functional Materials Technische Universität Berlin Berlin Germany; ^3^ Institute of Chemistry and Biochemistry Freie Universität Berlin Berlin Germany; ^4^ Division For Structure Analysis Bundesanstalt für Materialforschung und ‐prüfung (BAM) Berlin Germany; ^5^ Division For Surface and Thin Film Analysis Bundesanstalt für Materialforschung und ‐prüfung (BAM) Berlin Germany; ^6^ Institute of Chemistry Humboldt‐Universität Zu Berlin Berlin Germany

**Keywords:** gas physisorption analysis, N_4_ site, pore‐size artefact, tetrapyrrolic, ZnN_4_ site

## Abstract

Pore size analysis is essential for understanding and optimizing structure‐performance relations of functional carbon‐based materials including activated carbons, supercapacitor electrodes and atomically dispersed metal‐nitrogen‐doped carbon (M‐N‐C) catalysts. Pore size distribution (PSD) plots based on gas sorption porosimetry often show narrow micropores that are related to the adsorptive properties of named materials, which must be considered as artefacts arising from approximations in classical density functional theory (cDFT) models. By selectively preparing specific in‐plane functionalities using pyrolytic template‐ion (salt templating) reactions, we herein show that those apparent pores can be explained by preferential adsorption of the adsorbate molecules to specific in‐plane functionalities. Tetrapyrrolic Zn‐N_4_ sites are present in ZIF‐8 derived carbons, which are converted by Zn‐extraction into nitrogen‐doped carbons (NDC) comprising tetrapyrrolic H_2_N_4_ sites. DFT‐based calculation of adsorption energies allows the conclusive assignment of corresponding adsorption phenomena in comparative N_2_ vs. CO_2_ vs. Ar adsorption measurements additionally using Langmuir analysis. While the assignment of artefacts may improve the discussion of porosity, the determination of specific adsorption sites may be utilized as a valuable tool in materials science. Advanced models for the important material classes may allow accelerated progress in important energy‐related research fields.

## Graphical abstract

Gassorption analysis of in‐plane functionalized carbons reveals small, uniform ultramicropore signatures as potential artefacts of specific adsorption of the probe gases. Low‐pressure Langmuir fitting links these features to distinct functionalities via adsorption energies, enabling straightforward quantification of otherwise inaccessible surface‐site densities, demonstrated here for tetrapyrrolic ZnN_4_ and H_2_N_4_ sites.

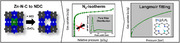

## 1 Introduction

**FIGURE 1:**
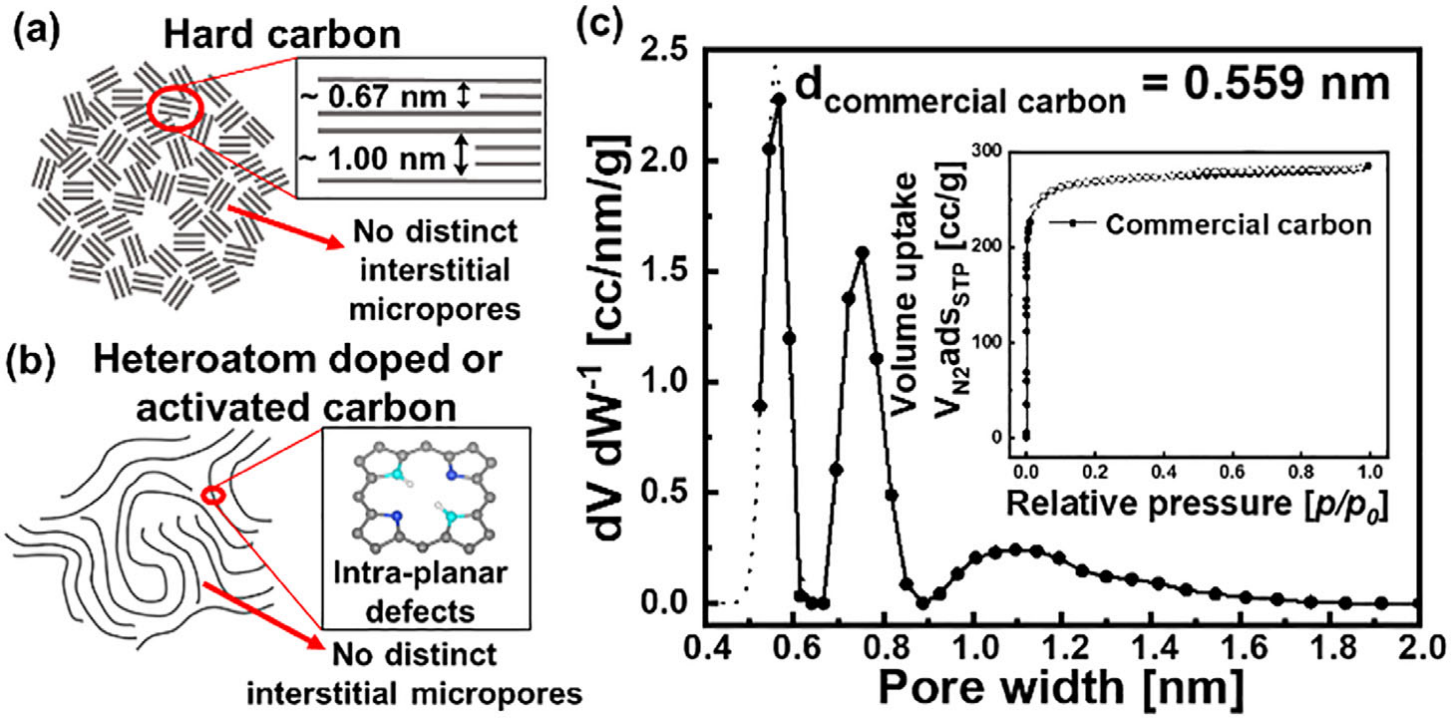
(a) Schematic illustrations of characteristic ultra‐ and supermicropores in a) hard/activated carbon and (b) amorphous/disordered or heteroatom doped carbon. (c) PSD plots of Hydraffin AA 8×30 with a very narrow peak, i.e. a very defined apparent ultramicropore, often observed for activated carbons. Dotted lines in black color show peak fitting using Gaussian function. In the inlet is the corresponding N_2_‐isotherm shown.

Gas‑sorption techniques are applied to characterize porous materials [1, 2, 3, 4, 5, 6, 7, 8], with advances in zeolite, metal‐organic‐framework (MOF), and covalent‐organic‐framework (COF) synthesis driving more sophisticated methods [9, 10, 11, 12, 13, 14]. Recently, the investigation of pores was critical to improve energy storage and conversion carbon‐materials such as, hard carbons as molecular sieves in anodes [15, 16], supercapacitors [17, 18], and M‐N‐Cs [19]. Understanding sorption in porous carbons is challenging due to their partially non‐graphitic, partially amorphous, highly disordered nature, unlike crystalline graphite [10, 20]. Activated carbons combine high capacity with strong adsorption; for example, N_2_ uptake at 77.4 K and low pressure indicates stronger adsorption compared to graphitic basal planes [7, 21, 22, 23, 24, 25, 26]. An ultrahigh surface area of 4800 m^2^ g^−1^ was obtained by a hypergolic reaction, a silica template and KOH activation [27]. Slit‑shaped pore models, reflecting graphene interlayer distances, explain distinct adsorption features when pore sizes match multiples of graphite's stacking distance (0.335 nm) [28]. The “house‑of‑cards” model describes non‐graphitizable carbons, so‐called hard carbons by nanocrystalline domains and interstitial pores, accounting for varied micropore and larger pore size distributions (PSDs) (Figure 1a) [29, 30, 31, 32, 33, 34, 35].

cDFT modeling accordingly links strong adsorption to specific pore sizes d_pore_, aiding structure–property interpretation and material optimization. Also other non‐graphitizable, non‐graphitic carbons, such as activated carbons are therefore tuned to precise micropore widths (≤ 2 nm) [36, 37, 38], including ultramicropores (< 0.7 nm) and supermicropores (0.7 – 2 nm), as measured by gas‐sorption porosimetry [39, 40]. However, highly microporous activated carbons, like other heteroatom‐rich carbons are more disordered in nature. They contain interstitial (ultra)micropores between distorted polyaromatic sheets and stacks (Figure 1b) [41], caused by defects and heteroatoms [42, 43]. Therefore, specific adsorption at narrow relative pressures is unlikely, since pore size and shape vary, leading to broad PSDs and unspecific adsorption. Nevertheless, commercial activated carbons show narrow apparent pores e.g. at 0.559 nm with a full width at half maximum (FWHM) of 0.0580 nm (Figure 1c) (details in the Supporting Information). This narrow FWHM is unexpected as for pores differing by one carbon layer, a FWHM ≥ 0.125 nm was shown [9]. cDFT, including nonlocal‐DFT (NLDFT) and quenched‐solid‐DFT (QSDFT), is widely used for simulating theoretical isotherms of predefined geometric pore models (e.g. slit, cylindrical and spherical), which forms the basis (kernels) for fitting experimentally measured isotherms for the determination of PSDs. These approaches approximate adsorbates as a continuous density, reducing computational cost and enabling corrections for non‐ideal factors like surface roughness without explicitly modelling them at the atomic level [21, 22]. They effectively describe monolayer, multilayer, and condensation processes by calculating the fluid‐density distribution that minimizes the grand‐canonical potential‐energy. However, in (ultra)micropores, this continuum assumption can yield unphysical density distributions, allowing fractional molecules and undersized pores compared to explicit methods such as electronic DFT. Although such pores are computational artefacts, their presence in PSDs signals adsorption stronger than expected from carbon–adsorbate interactions, suggesting potential strong adsorption sites.

Nitrogen adsorption–desorption analysis has revealed sharp peaks in the ultramicropore region for ZIF‐8‐derived carbons (ZIF‐8 ≙ specific zeolitic imidazolate framework), with significant N_2_ uptake (V = 0.277 cm^3^ g^−^^1^), apparent pore size < 0.6 nm, and FWHM ≈0.08 nm as reported by Mehmood et al. [44]. ZIFs, commonly used as carbon precursors for energy applications [44], exhibit large microporosity and offer tuneable nitrogen content and morphology [45, 46]. Carbonization at 750–900 °C yields M‐N‐Cs with in‐plane metal–N_4_ sites [47, 48], while Zn‐evaporation and aqueous work‐up create sub‐nanometer defects. Such materials are not simple adsorbents, as coordination sites and surface metal‐N_4_‐complexes act as preferential adsorption sites beyond standard models. Quantifying these catalytically active sites remains a major challenge [49].

**FIGURE 2:**
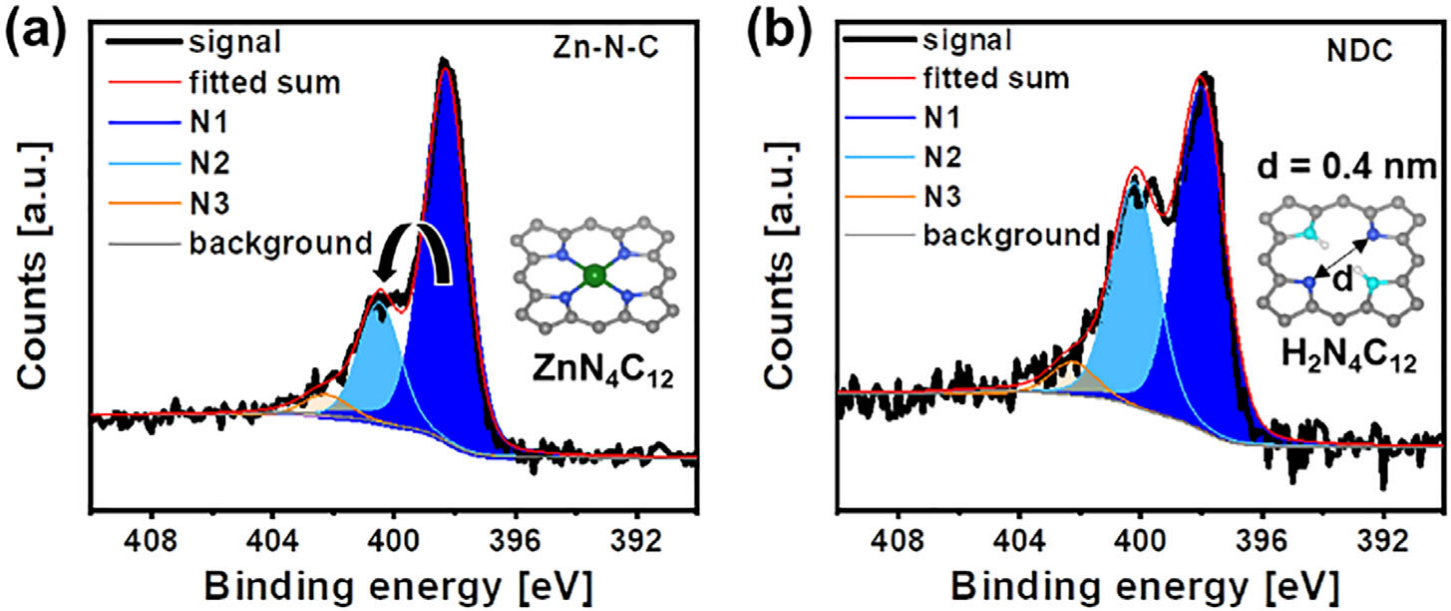
Deconvoluted XPS high resolution N 1s spectra of (a) Zn‐N‐C and (b) NDC samples. N1 peak corresponds to both pyridinic/imine and metal bound nitrogen (Zn‐N) sites, N2 and N3 peaks correspond to pyrrolic and graphitic nitrogen sites, respectively.

M‐N‐C surface metal complexes may exhibit various geometries such as square‐planar, distorted or penta‐coordinated [50] and structures such as tetrapyridinic [51], tetrapyrrolic [52] or even dual atom sites [50]. We showed that pyrolytic template‐ion reactions using Zn^2+^ as in ZIF‐derived carbons can result in selective formation of tetrapyrrolic ZnN_4_ sites (Figure 2a inset) [52], and justified by the higher spatial and energetic affinity of Zn^2+^ toward the formation of tetrapyrrolic N_4_ sites [53].

## 2 Results and Discussion

Here, the known synthesis and well‐characterized materials are used as model compounds to study gas‐sorption phenomena by adjusting the metal content and creating an ultramicropore, specific sorption site, respectively. The as‐prepared Zn‐N‐C contains 16.2 wt.% Zn and acid extraction of Zn^2+^ resulted in NDCs with 3.6 wt.% Zn remaining (Table S1). Extended‐X‐ray absorption fine structure spectroscopy (EXAFS) analysis of both materials confirmed a tetrapyrrolic Zn‐N_4_ coordination (Figures S1, S2 and Table S2) [52]. The similarity between the EXAFS profiles of Zn‐N‐C and NDC further implies homogeneity in the local structure of Zn between the leached and residual Zn‐sites, confirming the absence of inorganic Zn‐phases, also in agreement with the lack of Zn‐specific signals in the powder X‐ray diffractograms (PXRDs) (Figure S3). X‐ray photoelectron spectroscopy (XPS) of Zn‐N‐C and NDC was performed to monitor the transformation of ZnN_4_ to H_2_N_4_ sites (Figure 2b inset) by comparison with molecular analogues and peak fitting revealing a similar electronic situation in high‐resolution N1s spectra (Figure 2a,b, Table S3, details in Supporting Information) [54]. The main peak (dark blue) of Zn‐N‐C in Figure 2a is centered at 398.3 eV (N1), similar to the Zn‐N peak in Zn‐porphyrin [54]. Another peak (light blue) at 400.5 eV is assigned to pyrrolic N (N2). The removal of Zn^2+^ causes a change of intensity from the Zn‐N/imine functionality toward the pyrrolic functionality as in the molecular analogue confirming the formation of tetrapyrrolic H_2_N_4_ sites (Figure 2b). The corresponding tetrapyrrolic H_2_N_4_ site would therefore result in a cavity with a size in the ultramicropore range (N‐N distance of opposite N‐atoms of ∼ 0.4 nm (Figure 2b)). A reorganisation of nitrogen can be excluded as this would lead to more changes in the N 1s spectrum. Furthermore, the PXRDs (Figure S3a,b) do not show a change in graphitization excluding carbon etching. Therefore, it can be assumed that only Zn is removed, and the carbon framework remains unchanged, and the overall morphology remains.

**FIGURE 3:**
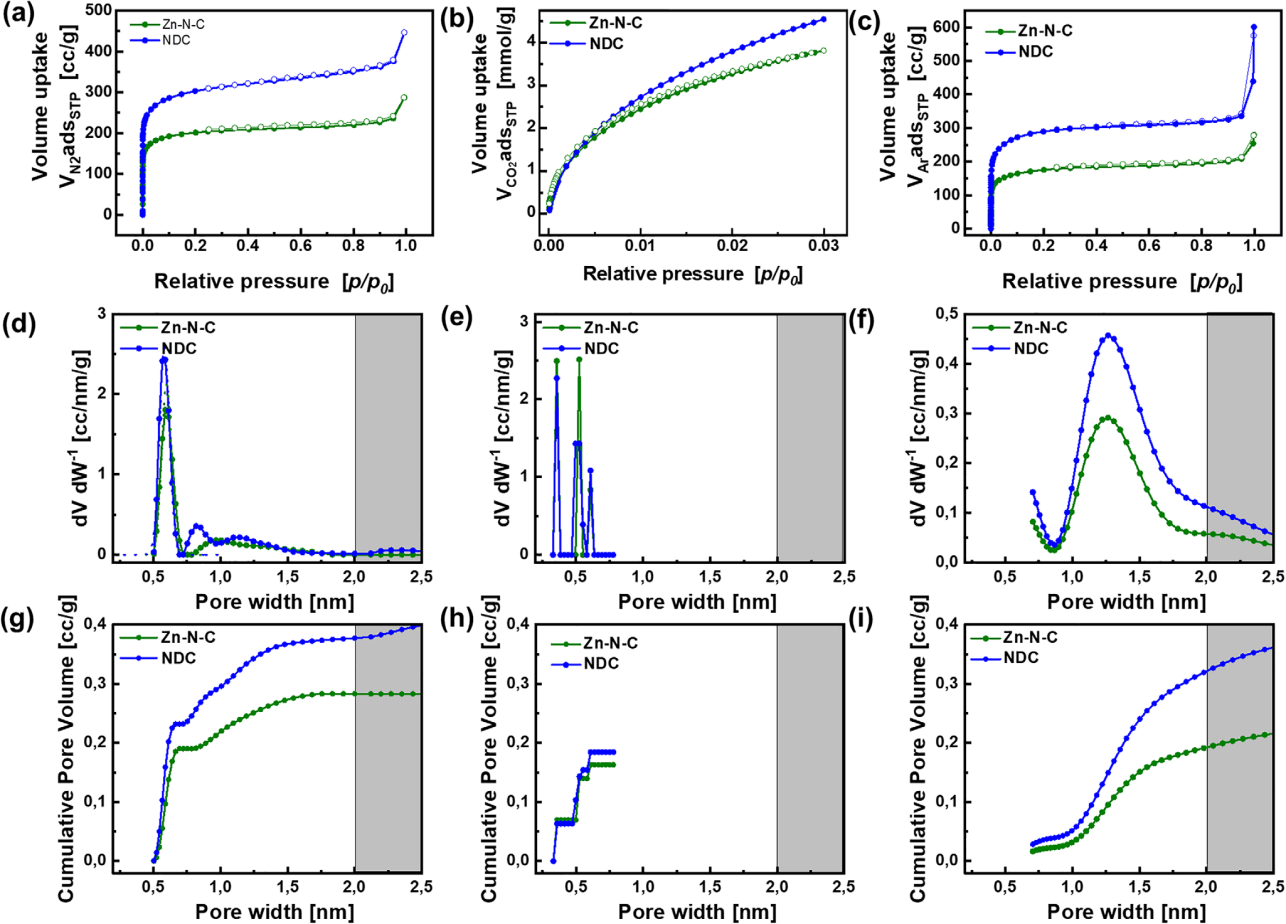
(a) N_2_ sorption isotherm plots of Zn‐N‐C and NDC samples measured at 77 K. (b) CO_2_ sorption isotherms measured at 273 K. (c) Ar sorption isotherms measured at 87 K. Pore size distribution plots of Zn‐N‐C and NDC, calculated from their respective N_2_ (d), CO_2_ (e) and Ar (f) adsorption isotherms by QSDFT and NLDFT method, respectively. Micropore volume plots of Zn‐N‐C and NDC, calculated from their respective N_2_ (g), CO_2_ (h) and Ar (i) adsorption isotherms by QSDFT and NLDFT method, respectively. Dotted lines in green and blue colors show peak fitting using Gaussian function. Details of QSDFT and NLDFT methods used for the calculation of pore size distributions and micropore volumes are given in the experimental section.

To investigate the origin of the distinct apparent micropores, gas‐sorption analysis was performed using N_2_ at T = 77.4 K, CO_2_ at T = 273 K and Ar at T = 87.3 K (Figure 3a‐c, raw data in Table S4). Both Zn‐N‐C and NDC show type I N_2_‐isotherms (Figure 3a) typical of microporous materials [39]. Zn^2+^‐extraction increases the apparent specific surface area (SSA_BET_) by 23%, possibly due to reduced specific weight after Zn removal. Surprisingly, the N_2_ uptake at lowest accessible relative pressures (p/p_0_ ≈ 10^−7^) is higher for the Zn‐N‐C than NDC, indicating for both gases a trend reversal (Figure S4a). This contradicts the generally higher gas uptake in the NDC compared to the Zn‐N‐C. The phenomenon is also counter intuitive if one considers the H_2_N_4_ sites as possible intraplanar ultramicropores (Figure S4a).

Similar to N_2_, the adsorption of type I CO_2_‐isotherm on Zn‐N‐C at the lowest relative pressures is more significant than on NDC (Figure S4b), despite higher total porosity and SSA for NDC.

In contrast to N_2_ and CO_2_, Ar‐sorption follows the expected trend in which NDC consistently exhibit higher specific gas uptake than Zn‐N‐C in the entire pressure range, i.e. without a reversal in the low pressure region (Figure S4c). The sensitivity of the low‐pressure uptake toward the probe gas (specifically its quadrupole moment) further justifies the exclusion of macroscopic origins for the reversal observed in N_2_/CO_2_, redirecting the focus toward microscopic interactions between adsorbate molecules and specific adsorption sites within the material. The IUPAC Technical Report 2015 on physisorption of gases suggests the use Argon, because it is less sensitive to specific adsorption phenomena. While this is followed e.g. in the MOF community, carbon materials are commonly analysed using N_2_ sorption. However, for both Zn‐N‐C and NDC the apparent SSA determination deviates for N_2_ and Ar, corroborating that specific adsorption is affecting the measurements. Apparent SSA values for the Zn‐N‐C are 566 m^2^ g^−1^ for Ar, and 750 m^2^ g^−1^ for N_2_. For the NDC the apparent SSA values are 995 m^2^ g^−1^ for Ar and 1082 m^2^ g^−1^ for N_2_. Interestingly, the N_2_ measurements seem to overestimate the values.

In the PSD plots (Figure 3d,e) the very narrow apparent ultramicropores (d < 0.7 nm) are found for N_2_ (FWHM ∼0.09 nm) and CO_2_ measurements (FWHM ∼0.04 nm), but not for Ar (Figure 3f). Such narrow peak widths would suggest atomically precise pore dimensions formed through the removal Zn ions from the Zn‐N‐C. The N_2_‐PSD suggests the formation of pores with diameters of 0.59 and 0.82 nm, while the CO_2_‐PSD suggests that the removal of Zn ions results in pores with a pore diameter of 0.61 nm on the costs of smaller pores with a diameter of 0.53 nm. Those unconclusive results have led the explanation that the narrow peaks are non‐physical and the peaks are artefacts of specific adsorption of the respective gas molecule to specific adsorption sites. The different properties of N_2_ and CO_2_ can explain the apparent pore size shifts better.

In line with the less or non‐interactive Ar, the Ar‐ PSDs only show broader supermicropores (0.7 nm < d < 2 nm) with a FWHM of ∼0.6 nm centred around 1.3 nm. Those pores are also present, but less prominent for the N_2_‐PSD and can reasonably be assigned to real micropores. Consequently, the offset between the isotherms of Zn‐N‐C and NDC can also be assigned to macroscopic differences, i.e. a gravimetric effect due removal of Zn and/or side reactions of the gaseous HCl treatment.

The cumulative pore volume plots also show the significant deviations in the porosity analysis between the different probe gases (Figure 3g‐i). The specific adsorption seems to facilitate pore filling resulting in increased apparent microporosity in the N_2_ analysis compared to Ar. The specific adsorption of N_2_ suggests a highly ultramicroporous material, although the microporosity almost entirely results from supermicropores (0.7< d < 2 nm). By CO_2_ only the ultramicropore volume region can be covered (Figure 3h), but the misinterpretation of specific adsorption sites as pores will also here falsely indicate a highly ultramicroporous material.

Summarising the experimental results, we can conclude that the reversal of gas uptake in the low pressure region has microscopic origins that both dependent on the quadrupole moment of the gas (CO_2_ > N_2_ > Ar) and the inherent specific adsorption site within the material, prompting further investigations into specific interactions between the gases and the imprinted ZnN_4_ and H_2_N_4_ site. Therefore, to identify adsorption sites, we studied their adsorption energies compared to the regular adsorption energy of the carbon support surface.

Using electronic DFT, we calculated the adsorption energies (E_ad_) of each probe gas (Ar, N_2_, CO_2_) on the tetrapyrrolic site (H_2_N_4_, ZnN_4_), and graphitic carbon (C_Gr_) cluster as reference (Table 1). The cluster model was chosen due to the highly defective carbon lattice (relative to honeycomb graphene) that is required to stabilize a tetrapyrrolic coordination, and the influence of cluster size on the CO_2_ adsorption energy was found to be < 0.01 eV (Table S7). We observed that for all three gases, the adsorption on ZnN_4_ site is stronger than on graphitic carbon or H_2_N_4_ site. However, the deviation from C_Gr_ increases with increasing quadrupole moment of the probe gas (Ar < N_2_ < CO_2_). We attribute this trend to the high charge polarization that arises from the ionic binding character at the ZnN_4_ site, with the Zn atom assuming a net natural bond orbital (NBO) charge of +1.6 e [53]. An analysis of the NBO charge and bonding geometry of adsorbed gases at the ZnN_4_ site further indicates dative bonding character with significant polarization of electron density toward Zn (Supporting information, Table S6). As a result, gases with higher quadrupole moment (CO_2_ > N_2_ > Ar) show higher difference in adsorption energies at ZnN_4_ sites relative to C_Gr_. We further note that the relatively large quadrupole moment of CO_2_ also seems advantageous for its adsorption to the H_2_N_4_ sites (relative to graphitic carbon) as the optimized adsorption geometry aligns with the direction of the N‐H bonds, suggesting quadrupole‐dipole interactions. However, the quadrupole‐dipole interaction at H_2_N_4_ is still weaker than the quadrupole‐ion interaction at the ZnN_4_ site, leading to a weaker adsorption energy on the former (ΔΔE_ad_ = 0.035 eV, ΔΔG_ad_ = 0.028 eV). In contrast to CO_2_, the adsorption of Ar relative to C_Gr_ is marginally stronger on ZnN_4_, and weaker on H_2_N_4_. As deviations to C_Gr_ are small, any microscopic interactions with Ar would effectively be masked by the majority adsorption at the carbon sites, thereby explaining the lack of artefacts in the Ar‐PSD and the higher initial Ar uptake in the Ar‐isotherm. Overall, based on the deviation of adsorption energies from graphitic carbon, CO_2_ with its high quadrupole moment would be ideal for probing microscopic feature embedded active sites, but conversely results erroneous mesoscopic properties if interactions with specific features are neglected from analysis. In contrast, the inert and non‐quadrupolar Ar is more suitable for artefact‐free macroscopic analysis of pore sizes and surface areas.

**TABLE 1:** Zero‐point energy and counterpose‐corrected adsorption Energies of Ar, CO_2_ and N_2_ on graphitic carbon C_Gr_, tetrapyrrolic H_2_N_4_ and ZnN_4_ sites. ΔG are approximated with vibrational partition functions at the respective experimental measurement temperatures of the probe gases.

Gas	Site	ΔE_ad_ [eV]	ΔG_ad_ [eV]
Ar	C_Gr_	−0.056	+0.048
Ar	H_2_N_4_	−0.038	+0.060
Ar	ZnN_4_	−0.064	+0.035
N_2_	C_Gr_	−0.078	+0.034
N_2_	H_2_N_4_	−0.081	+0.030
N_2_	ZnN_4_	−0.116	−0.003
CO_2_	C_Gr_	−0.162	+0.187
CO_2_	H_2_N_4_	−0.203	+0.164
CO_2_	ZnN_4_	−0.238	+0.136

**FIGURE 4:**
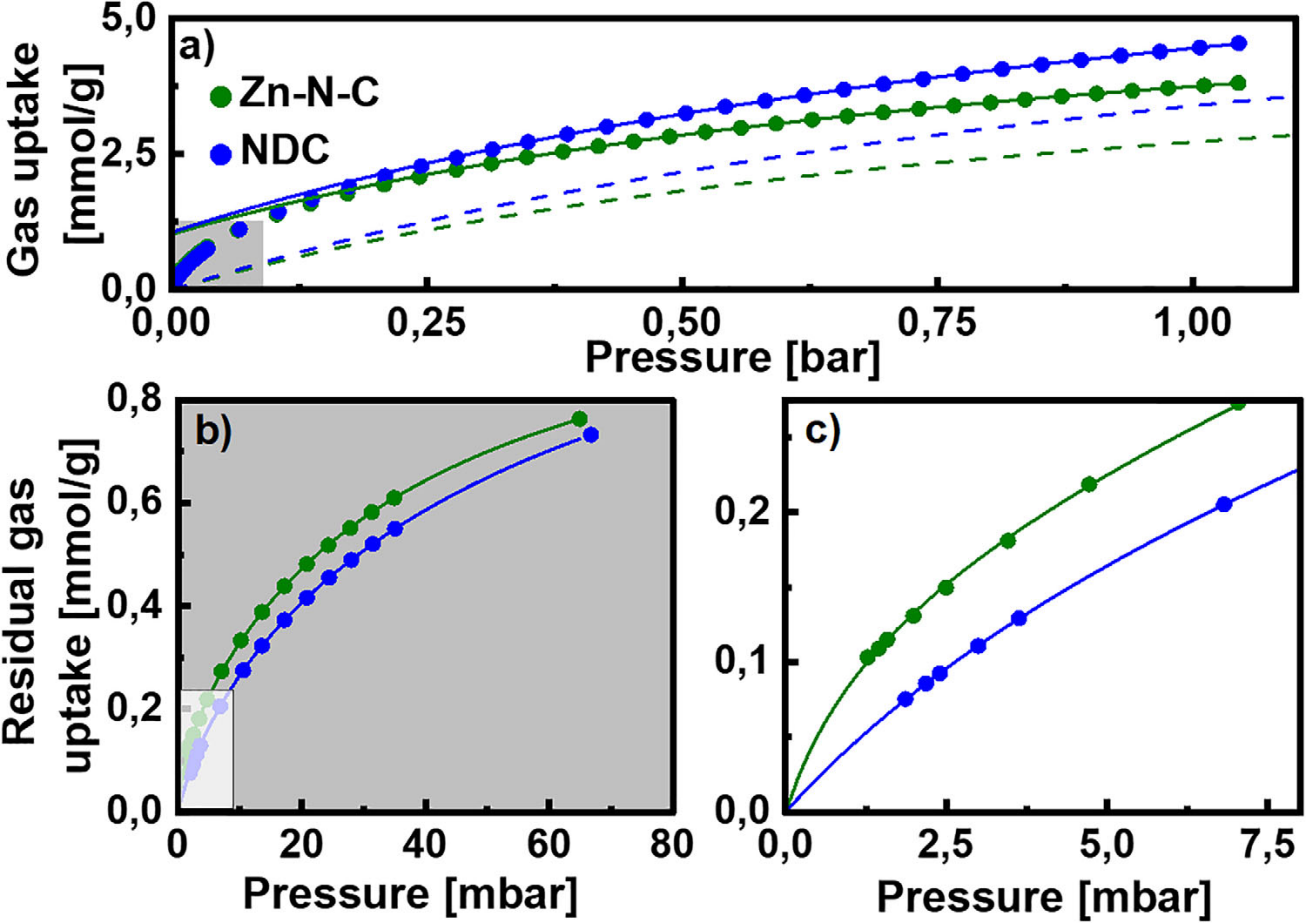
(a) Raw CO_2_ isotherm plots (blue and green circles) for (a) the entire measured pressure range with (b) plot of residual adsorption quantity as defined in equation 3 (p < 0.08 bar). The solid lines in (a) are the fitted Langmuir equation with parameters given in Table 2 and the dotted lines represent the hypothetical adsorption profile of the weak binding site (N_C_) if the stronger binding sites were not present. (b) is the inset of the light grey area in (a) and (c) is the inset of the deep grey area in (b).

Since both ZnN_4_ and H_2_N_4_ sites show higher adsorption energies compared to C_Gr_ (neither Ar, N_2_ nor CO_2_ fill the cavity of the H_2_N_4_ site, Figure S5), indicating they are indeed preferential adsorption sites with binding energies in the ultramicropore range and can explain the narrow peaks in the PSD plots as artefacts of specific adsorption to surface sites. Since the PSD is already fitted to assumed pore structures, it cannot directly provide information on specific adsorption energies. Therefore, we explore an alternative analysis approach using the multi‐Langmuir analysis to obtain structure–adsorption correlations for improved active site characterization. For both materials, three different adsorption phenomena could be identified upon Langmuir fitting (details on the fitting procedure in Supporting Information). From Figure 4a, it is evident that the fitted Langmuir isotherm deviates at the lower pressure regions (p < 0.1 bar), and the mismatch can only be justified by the presence of stronger binding sites.

Contributions of the weak binding sites at lower pressure were excluded, by subtracting their hypothetical Langmuir isotherm if the stronger binding sites were absent (i.e. *N*_C_ in Equation S2, dotted lines in Figure 4a) from the measured adsorption quantities to obtain the adsorption profile of the stronger binding sites. This quantity is plotted against absolute pressure as shown in Figure S6 and Figure 4. From Figure S6a, the residual plot is approximately constant at higher pressure regions, confirming a good fit for the weak binding site, while a steep increase at low pressures indicates stronger binding sites. For p < 0.08 bar, we fitted the residual adsorption to a Langmuir model with two active sites (Equation S3) of differing adsorption energies. We note that a single‐site Langmuir equation fit to the residual plot was attempted but is unable to produce a good fit (Figure S6b), whereas the fitting quality improved with a double‐site Langmuir (Equation S4). The fitting reveals a second adsorption site with similar Langmuir parameters (Table 2) and quantities in both materials (K_eq,2_ ≈ 20, N_max,2_ ≈ 1 mmol g^−1^), possibly attributed to the carbon/nitrogen functionalities or pore structures. Interestingly, the third set of Langmuir parameters shows significant deviations in the equilibrium constant between Zn‐N‐C (*K*_eq,3_ = 760, ΔG^0^ = −0.16 eV) and NDC (*K*_eq,3_ = 165, ΔG^0^ = −0.12 eV). This can be attributed to the difference in initial curvature (K_eq_) required to explain the higher and faster initial volume uptake in Zn‐N‐C compared to NDC (Figure 4b). Although our analysis here emphasizes the strongest binding site within the material (i.e. adsorption site 3), we also note that all three equilibrium constants derived from the multi‐Langmuir analysis are consistently higher for Zn‐N‐C than for NDC despite generally higher gas uptake (N_max_) in the latter. Therefore, it is possible that specific adsorption sites may also have influenced the curvature of the adsorption profile at higher pressures and potentially lead to underestimated pore sizes, although deconvoluting these effects from the majority adsorption on carbon remains a challenge for future studies.

**TABLE 2:** Fitted Langmuir parameters based on Equations S3 and S4 for CO_2_ adsorption isotherms.

	Zn‐N‐C	NDC	Site
N_0,1_ (mmol/g)	1.03 ± 0.01	1.07 ± 0.02	C_support_
N_max,1_ (mmol/g)	5.35 ± 0.02	7.66 ± 0.06	
K_eq,1_	1.04 ± 0.02	0.80 ± 0.02	
ΔG^0^_1_ (eV)	≈0	≈0	
N_max,2_ (mmol/g)	0.995 ± 0.006	1.085 ± 0.002	C_pores_
K_eq,2_	26.3 ± 0.7	16.2 ± 0.2	
ΔG^0^_2_ (eV)	−0.0769 ± 0.0007	−0.0655 ± 0.0003	
N_max,3_ (mmol/g)	0.138 ± 0.006	0.184 ± 0.004	ZnN_4_/ H_2_N_4_
K_eq,3_	760 ± 60	165 ± 4	
ΔG^0^_3_ (eV)	−0.156 ± 0.002	−0.1201 ± 0.0006	

As adsorption strength generally correlates inversely with apparent pore width from PSDs, the unrealistically small “pores” are likely to correspond to specific adsorption sites. Notably, the difference in Langmuir‐fitted adsorption free energy of ΔΔG^0^ = 0.036 eV between Zn‐N‐C and NDC is consistent with the DFT‐predicted differences in the CO_2_ adsorption energies at these sites ΔΔE = 0.035 eV, ΔΔG^0^ = 0.028 eV) at tetrapyrrolic‐N_4_ sites (Table 1). Furthermore, the difference between ΔG^0^_2_ and ΔG^0^_3_ within the same material (ΔG^0^_2_ – ΔG^0^_3_ = 0.08 eV for Zn‐N‐C and ΔG^0^_2_ – ΔG^0^_3_ = 0.055 eV in NDC) approximately agrees with the DFT‐predicted differences between respective active sites and C_Gr_ (ΔΔE_ad_ = 0.076 eV for Zn‐N_4_ vs C_Gr_; ΔΔE = 0.041 eV for H_2_N_4_ vs C_Gr_). This suggests that ΔG^0^_2_ and ΔG^0^_3_ may reflect different binding sites within the same confinement environment. Finally, we note that the maximum adsorption capacity *N*_max,3_ (Table 2) at these sites lies in the same order of magnitude as the accessible site densities in Fe‐N‐Cs derived from pyrolyzed ZIF‐8 materials (0.08 – 0.13 mmol g^−1^) [44], highlighting the potential of this analytical approach in approximating accessible active sites upon calibration.

## 3 Conclusion

For activated carbons and other non‐graphitic carbon materials, such as heteroatom doped carbons and atomically‐dispersed M‐N‐Cs, gas‐sorption analysis may show apparent micropores with peak widths below the empirical threshold of FWHM = 0.125 nm [9], arising from in‐plane functionalities (referred to as defects). The herein used salt‐templating synthesis to prepare ion‐templated carbons and atomically dispersed M‐N‐Cs, allowed us to controllably prepare such in‐plane functionality. These known sites were used to assign the narrow peaks to preferential adsorption sites. This discovery allowed us to develop a method to further analyze the low‐pressure range by the existing Langmuir analysis and it is recommended to mark those peaks in reported PSDs e.g. with an *asterisk* as artefact. Accordingly, the conventionally calculated micropore or ultramicropore volumes should be reported as apparent micropore and apparent ultramicropore volumes. Generally, for atomically‐dispersed M‐N‐Cs and related NDCs it is recommended to report SSA_BET_ and TPV from Ar‐sorption measurements to avoid overestimation due to specific adsorption. The possibility to detect and possibly quantify specific surface sites and in‐plane functionalities in simple gas‐adsorption measurements can turn it into a powerful tool for the optimization of properties of carbon‐based materials in energy storage and conversion applications (precious‐metal free catalysis, carbon‐based anode materials and general sorption applications) by developing new kernels. Additionally, the findings may also guide synthesis strategies for the development of doped porous materials by deconvoluting influences from morphological structural properties like porosity and SSA from chemical structural properties like active site geometry and concentration, both of which are crucial for functional performance.

## Author Contributions

**Simon W. J. Dietzmann**: Investigation, Formal analysis, Methodology, data analysis, experimental work on the synthesis and characterization (N_2_, CO_2_‐isotherms, XAS). **Asad Mehmood**: Investigation, Formal analysis, Methodology, Supervision, involved in the conceptualization, the experimental planning and analysis as well. **Jian Liang Low**: Formal analysis, Methodology, Supervision, involved in the conceptualized and performed DFT calculations, highly involved in the overall data analysis. **Shu‐Han Wu**: Investigation, Methodology, measuring N_2_‐isotherms. **Carsten Prinz**: Investigation, measuring CO_2_ isotherms. **Ana Guilherme Buzanich**: Investigation, Formal analysis, Methodology, Supervision, measuring XAS. **Jörg Radnik**: Formal analysis, Methodology, carried out XPS analysis. **Paul A. Appel**: Investigation, Methodology, measuring N_2_‐isotherms and managing instrumentation. **Franziska Emmerling**, Supervision and  revised the manuscript. **Tim‐Patrick Fellinger**: Investigation, Formal analysis, Methodology, Supervision, Conceptualization, Funding acquisition, conceptualized and managed the project, was involved in experimental planning and data analysis, and wrote the manuscript.

## Conflicts of Interest

The authors declare no conflicts of interest.

## Supporting information




**Supporting File**: advs76048‐sup‐0001‐SuppMat.pdf.

## Data Availability

The data that support the findings of this study are available from the corresponding author upon reasonable request.
