# Supplementary material for: A Pore or not a Pore? Understanding Pore Size Distributions of Non‐Graphitic Carbon and Atomically‐Dispersed M‐N‐C Materials
Source: Adv Sci (Weinh). 2026 Jun 12;13(50):e76048. doi: 10.1002/advs.76048 (PMC13336846; doi:10.1002/advs.76048)
Supplement: Supplementary file 1 [file ADVS-13-e76048-s001.pdf]

SUPPORTING INFORMATION

"A Pore or Not a Pore? Understanding Pore Size Distributions of Non-Graphitic Carbon and Atomically-Dispersed M-N-C Materials"

Simon W. J. Dietzmann,[a,d] Asad Mehmood,[a] Jian Liang Low,[a,e] Shu-Han Wu,[a,d] Carsten Prinz,[b] Ana Guilherme Buzanich,[b] Jörg Radnik,[c] Paul A. Appel,[a,f] Franziska Emmerling,[b] Tim-Patrick Fellinger*[a]

[a] Division for Electrochemical Energy Materials, Bundesanstalt für Materialforschung und -prüfung (BAM), Unter den Eichen 44-46, 12203 Berlin, Germany
E-mail: tim-patrick.fellinger@bam.de

[b] Division for Structure Analysis, Bundesanstalt für Materialforschung und -prüfung (BAM), Richard-Willstätter-Strasse 11, 12489 Berlin, Germany

[c] Division for Surface and Thin Film, Bundesanstalt für Materialforschung und -prüfung (BAM), Unter den Eichen 44-46, 12203 Berlin, Germany

[d] Department of Chemistry, Functional Materials Technische Universität Berlin, Hardenbergstr. 40, 10623 Berlin, Germany

[e] Institute of Chemistry and Biochemistry, Freie Universität Berlin, Arnimalle 22, 14195 Berlin, Germany

[f] Institute of Chemistry, Humboldt-Universität zu Berlin, Brook-Taylor-Str. 2, 12489 Berlin, Germany

DOI: 10.1002/anie.2016XXXXX

SUPPORTING INFORMATION

Table of Contents

1. Experimental Procedures
   1.1. Synthesis of Zn-N-C and NDC
   1.2. Physiochemical characterizations
   1.3. Active Site Modelling
   1.4. Multi-Langmuir Analysis
   1.5. Gas-sorption data
   1.6. Models and Results of DFT calculations:

2. **Table S1.** The Zn content obtained from ICP-OES analysis.

3. **Figure S1.** XANES spectra of Zn-N-C and NDC at the Zn K-edge (for NDC residual Zn can be measured).

   Figure S2. Experimental (phase-corrected) Fourier transform and best fit EXAFS model of the Zn K-edge EXAFS spectra.

   Table S2. Structural information obtained from EXAFS.

4. **Table S3**. XPS fitting parameter for the N1s spectra.

5. **Figure S*3*.** PXRD-spectra of Zn-N-C and NDC.

6. **Figure S4**. Initial gas-uptakes of Zn-N-C and NDC in a) N_2-isotherm, b) CO_2-isotherm and c) Ar-isotherm.

Table S4. Raw-experimental data of the N_2, CO_2 and Ar isotherms of Zn-N-C and NDC.

7. **Figure S5.** Adsorption geometries of Ar (top), N_2 (middle) and CO_2 (bottom) on graphitic carbon cluster C_{Gr} (left), H_2N_4 site (middle) and ZnN_4 site (right) optimized with M06-2X functional, def2-SVP basis and D3(BJ) dispersion correction.
8. **Figure S6.** (a) Plot of residual CO_2 adsorption quantities obtained upon subtraction of the theoretical isotherm of the main (weak) adsorption from the raw isotherm for (a) the entire measured pressure range and (b) at low pressures (P < 0.08 bar). The line in (b) represents the best-fit to a single-site Langmuir equation.

9. **Figure S7.** PSD derived starting at $p/p0=10^{-5}$ for Zn-N-C and NDC.

10. **Table S5.** Elemental composition of the Zn-N-C and NDC samples

11. Supporting References

1. Experimental Procedures

1.1. ***Synthesis of Zn-N-C and NDC:*** Zn-N-C was obtained by pyrolyzing 3 g of commercial ZIF-8 (zinc 2-methylimidazolate, Basolite©, BASF) in an alumina boat at 900 °C for 2 h under constant N_2 flow in a tube furnace. A heating rate of 3 K min^{-1} was used to reach the set temperature of 900 °C. After carbonization, the furnace was cooled down to room temperature passively and Zn-N-C product was collected. On average the Zn-N-C product was around 1.3-1.5 g (42-50% yield).

The NDC was obtained by extracting Zn from Zn-N-C using HCl gas as reported the literature.[1] 1 g of Zn-N-C was dispersed in an alumina crucible and heated to 800 °C at a heating rate of 10 K/min and maintained at this temperature for 1.5 h under constant Ar flow. At 750 °C, HCl gas was added for 1 h. Subsequently, the quartz tube was convectively cooled to ambient temperature and the black powder was subjected to an overnight wash in 1 L of ultrapure water (MilliQ 18.2 MΩ cm) at 80°C. The resulting dispersion was filtrated, and the black solid was dried at 250°C for 12 h and yielded 850 mg (85%) of NDC.

1.2. ***Physicochemical characterization*:** Nitrogen and Argon sorption measurements were performed using an Anton Paar (Quantachrome) Autosorb iQ-C-MP instrument after outgassing of the samples for 12 h at 250 °C under turbopump vacuum. Specific surface areas were determined using the Micropore BET Assistant supplied by ASiQwin software. Nitrogen sorption measurements were completed at 77.4 K and Ar sorption measurements were performed at 87.3 K. The pore size distributions were calculated with the quenched-solid density functional theory (QSDFT) method (slit/cylindric/sphere pores, adsorption model). For comparison, the commercial carbon Hydraffin AA 8x30 from Donau Carbon GmbH was measured. Carbon dioxide sorption was measured on a TriStar II 3020 Version 3.02 from Micromeritics at 273.15 K. 80-100 mg material per sample was used and degassed for 12 h at 250°C. At the applied temperature of the CO_2 measurements, calculations of the pore size distribution of micropore systems up to 0.8 nm (p/p0 = 0.03) can be performed using DFT, based on either the adsorption or desorption isotherm. The adsorption branch was used and fitted with CO_2@273 K carbon slit pores, using non-localized density functional theory (NLDFT). X-ray photoelectron spectroscopy (XPS) measurements were performed with an ULVAC-PHI Quantes photoelectron spectrometer manufactured by Ulvac-PHI (Chanhassen, MN, USA). XPS spectra were recorded using monochromatized aluminum K_α radiation for excitation, at a pressure of approximately $5 \cdot 10^{-9}$ mbar. The electron emission angle was 45° and the source-to-analyzer angle was 45°. The binding energy scale of the instrument was calibrated following a PHI procedure which uses ISO 15472 binding energy data. C 1s at 284.5 eV was used for the charge correction. For peak fitting a sum Gaussian-Lorentzian function was used. A modified Shirley background was used for background correction. Details about the analysis: Polzonetti et al. investigated their electronic structure using XPS and showed that the Zn(II)-coordinated complex displays only one N1s core level peak at 398.0 eV, indicating electronic equalization of all four N-atoms coordinating the Zn-ion, whereas the metal-free analogue showed the expected two core level peaks for the protonated pyrrolic and imine nitrogen at binding energies of 399.5 and 397.5 eV.[2] Both spectra show a minor contribution at 402.3 eV, assigned to graphitic N-species as additional functionalities of the materials (N3). Moreover, we observe a deviation from the expected 1:1 ratio in the relative intensities of protonated and unprotonated pyrrolic peaks (imines), compared to porphyrins, presumably due to additional non-macrocyclic imine, pyrrolic functionalities, remaining ZnN_4 sites and existing H_2N_4 sites within the Zn-N-C scaffold. X-ray absorption spectroscopy (XAS) containing XANES and EXAFS was performed at the BAMline at BESSY-II storage ring (Helmholtz Center Berlin) in continuous transmission mode and analyzed with DEMETER software package.[3] XRD data were recorded with a Bruker diffractometer with Cu Kα radiation (λ = 1.54060, 50 kV, 30 mA, germanium (111)) and analysed with Match!. Elemental Analysis (CHNS/O) was performed on a ThermoFisher FlashSmart 1120 and three measurements were averaged. ICP-OES was performed on an Agilent 5800.

1.3. ***Active Site Modelling*:** Electronic DFT calculations were performed with spin-unrestricted Kohn–Sham DFT using the program package TURBOMOLE.[4] We applied the hybrid M06-2X functional[5] with Grimme's D3(BJ) dispersion correction with Becke Johnson damping (https://doi.org/10.1063/1.3382344, https://doi.org/10.1002/jcc.21759) for the modelling of non-covalent interactions during the adsorption process. Each cluster was geometrically optimized with the def2-SVP basis in various multiplicities, and a subsequent single point calculation was performed using the def2-TZVP basis for the multiplicity with the lowest energy.[6] Zero-point energy (ZPE) correction was performed through vibrational frequency analysis in the def2-SVP basis. Counterpoise correction in the def2-TZVP basis was performed in the optimized geometry of the adsorbed state for each cluster/adsorbate pair.[7] Electronic steps were converged to 10^{-7} au in the total energy and 10^{-4} au in the orbital

energies, while geometric steps were converged to 10^{-6} au in the total energy and 10^{-3} au in the geometric gradient. We used a planar tetrapyrrolic $MN_4C_{96}H_{24}$ cluster model for the modelling of the tetrapyrrolic sites.[8] Atomic charge populations were obtained with the natural population analysis. Atomic structures were visualized with VESTA.[9]

For the calculation of adsorption free energies, the absolute free energies of gases are calculated with

$$G_{\text{gas}} = E_{\text{DFT}} + ZPE + H_{\text{gas}} - TS_{\text{NIST}}$$

where *ZPE* is the zero-point energy, H_{gas} is the standard absolute enthalpy of ideal gases ($H_{\text{linear gas}}$ = 3.5 k_BT for CO_2 and N_2, $H_{\text{monoatomic gas}}$ = 2.5k_BT for Ar), *T* is the temperature at which the gas sorption study is performed and S_{NIST} is the standard entropy obtained from the NIST database. The free energies of the pristine and adsorbed clusters are calculated with

$$G_{\text{cluster}} = E_{\text{DFT}} + ZPE + F_{\text{vib}}$$

where *ZPE* and F_{vib} are derived upon frequency analysis of the cluster

$$F_{\text{vib}} = \sum_i k_B T \ln\left(1 - exp\left(\frac{-hc\nu_i}{k_B T}\right)\right)$$

where ν_i is the vibrational wavenumber, k_B is Boltzmann's constant, *h* is the Planck's constant and *c* is the speed of light. Free energies of adsorption are defined as the energy difference between the adsorbed cluster $G_{\text{M-X}}$ and the respective adsorbate-free cluster G_{M} and ideal gas G_{X}

$$\Delta G_{\text{ad}} = G_{\text{M-X}} - G_M - G_{\text{X}} - BSSE$$

where the *BSSE* is the basis set superposition error that is calculated in the adsorbed geometry (M-gas)

$$BSSE = E_{\text{M}}^{\text{M-X}} + E_{\text{X}}^{\text{M-X}} - E_{\text{M}}^{\text{M}} - E_{\text{X}}^{\text{X}}$$

where notation $E_{\text{fragment}}^{\text{basis}}$ is used. E_{M}^{MX} and E_{X}^{MX} are respectively the DFT energies of the pristine cluster M and gas molecule X calculated in the full basis of the M-X. E_{M}^{M} and E_{X}^{X} are the DFT energies of the pristine cluster M and gas molecule X in their own defined basis.

1.4. **Multi-Langmuir Analysis:** The Langmuir adsorption model assumes no interaction among adsorbed species and a 1:1 ratio between the adsorbate and active site, both could be fulfilled by tetrapyrrolic-N_4 sites in M-N-Cs (equation S1). For analyzing the weakest binding site at higher pressures (*p* > 0.3 bar) (Fig. 4), corresponding to N_{C}, we applied a single-site Langmuir equation with an additional factor N_{res} to account for other (stronger binding) active sites:

$$N = N_{\text{C}} + N_{\text{res}} = \frac{N_{\text{max,1}} K_{\text{eq,1}} p}{1 + K_{\text{eq,1}} p} + N_{\text{res}}(p) \qquad \text{(S1)}$$

where N_{max} is the theoretical saturation quantity of adsorbate at this site and K_{eq} is the equilibrium constant of adsorption. We further assume that stronger binding sites contributing to N_{res} are saturated at these pressures (equation S1, S2) and thus contribute a mere constant to equation S1 ($N_{\text{res}} \approx$ constant for *p* > 0.3 bar). This assumption seems realistic for CO_2-sorption isotherms, since H_2N_4 and ZnN_4 sites binding more strongly to adsorbates than graphitic carbon (Table 1). Nevertheless, due to their sheer quantities relative to specific adsorption sites, these weak binding sites dominate the adsorption isotherm, so their contributions cannot be neglected even at low pressures. Therefore, we subtract its hypothetical isotherm from the measured isotherm to obtain residual isotherm for the stronger adsorption sites.

$$N_{\text{res}}(p) = N - N_{\text{C}} = N - \frac{N_{\text{max,1}} K_{\text{eq,1}} p}{1 + K_{\text{eq,1}} p} \qquad \text{(S2)}$$

This residual isotherm is subsequently fitted to a double-site Langmuir equation to obtain the corresponding saturation quantity and equilibrium constants for each site.

$$N_{\text{res}}(p) = \frac{N_{\text{max,2}} K_{\text{eq,2}} p}{1 + K_{\text{eq,2}} p} + \frac{N_{\text{max,3}} K_{\text{eq,3}} p}{1 + K_{\text{eq,3}} p} \qquad \text{(S3)}$$

Finally, the standard Gibb's free energy of adsorption at each site *i* is calculated from the respective equilibrium constants from the Langmuir fitting (Equations S1 and S3).

$$\Delta G^0{}_i = -RT \ln K_{\mathrm{eq,i}} \qquad \text{(S4)}$$

We note that the equilibrium constant K_{eq} is referenced to standard temperature and pressure of CO_2 (T = 273 K, p = 1 bar) as opposed to the reference to saturation CO_2 pressure that is conventionally used for pore-size distribution analysis.

$$K_{\mathrm{eq}} = \frac{p}{1\ \mathrm{bar}} \qquad \text{(S5)}$$

2 ICP-OES

Table S1. The Zn content obtained from ICP-OES analysis.

Sample	Zn wt.%
Zn-N-C	16.2
NDC	3.6

3 XAS

XANES

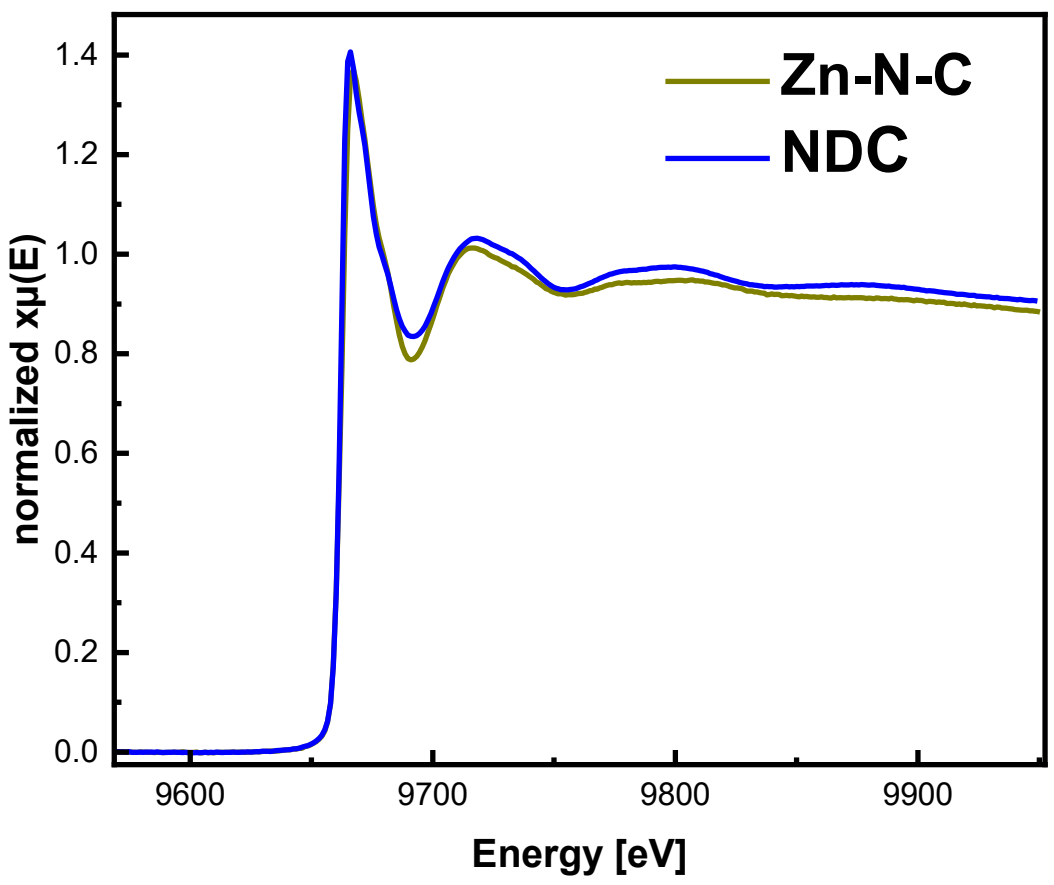

Figure S1. XANES spectra of Zn-N-C and NDC at the Zn K-edge (for NDC residual Zn can be measured).

EXAFS

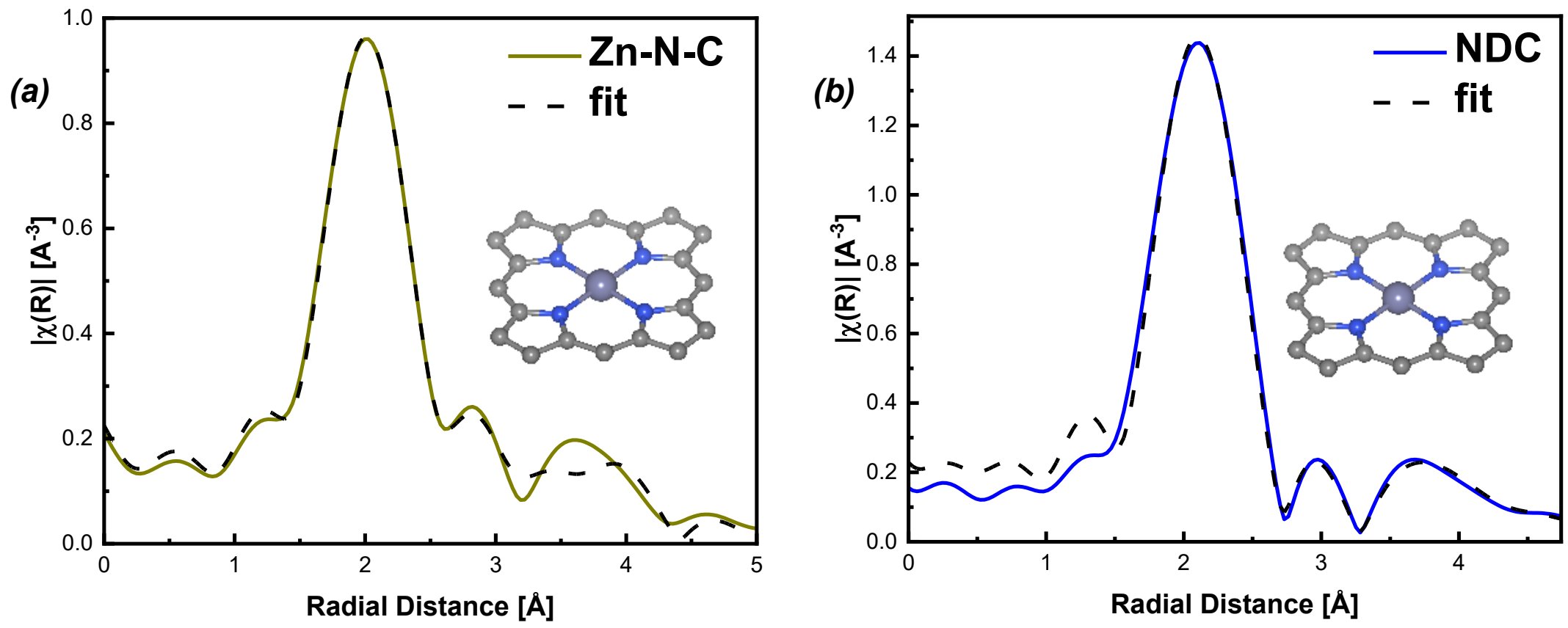

Figure S2. Experimental (phase-corrected) Fourier transform and best fit EXAFS model of the Zn K-edge EXAFS spectra of a) Zn-N-C and b) NDC Zn K-edge EXAFS spectra. The model used for Zn-N-C is displayed in the inset (Light blue = Zn; blue = N; gray = C; white = H).

Table S2. Structural information obtained from EXAFS by fitting the nearest coordination shells around the Zn atom in the samples with model $ZnN_4C_{96}H_{24}$: degeneracy of the scattering path (N), interatomic distance from the fit (R) and from the model (Reff), and Debye-Waller factor (σ^2). The goodness-of-fit parameter is indicated by the R-factor. The EXAFS curves were phase corrected before extraction of structural information.

Zn-N-C							
S_0^2 = 1.22; ΔE_0 = 2.68							
Scattering path	N	R (Å)	Reff (Å)	σ^2 (Å^2)	ΔR	R-factor	
Zn-N	4	2	2.01	0.0094	-0.004	0.01	Single-scattering
Zn-C	8	3.03	3.04	0.0352	-0.004		Single-scattering
Zn-N-C	16	3.21	3.21	0.0352	-0.004		Triangle scattering
Zn-N-C-N	8	3.38	3.38	0.0352	-0.004		Dog-leg-scattering
Zn-C	4	3.45	3.45	0.0302	-0.004		Single-scattering
Zn-C	8	4.26	4.26	0.0302	-0.004		Dog-leg-scattering
Zn-N-C	16	4.29	4.29	0.0302	-0.004		Triangle scattering
Zn-N-C-N	8	4.5	4.31	0.0302	0.2		Triangle scattering
Zn-C-C	16	4.35	4.36	0.0187	-0.004		Triangle scattering
Zn-C	4	4.90	4.90	0.0187	-0.004		Single-scattering
Zn-C-C	8	4.66	4.90	0.0187	-0.2		Forward-scattering
NDC							
S_0^2 =1.34; ΔE_0 = 7.93							
Scattering path	N	R (Å)	Reff (Å)	σ^2 (Å^2)	ΔR	R-factor	
Zn-N	4	2.06	2.01	0.0037	0.05	0.0048	Single-scattering
Zn-C	8	3.10	3.04	0.0151	0.06		Single-scattering
Zn-N-C	16	3.21	3.21	0.0151	0.005		Triangle scattering

Zn-N-C-N	8	3.44	3.38	0.0037	0.06		Dog-leg-scattering
Zn-C	4	3.46	3.45	0.007	0.005		Single-scattering
Zn-C	8	4.27.	4.26	0.007	0.005		Dog-leg-scattering
Zn-N-C	16	4.34	4.29	0.0151	0.05		Triangle scattering
Zn-N-C-N	8	4.32	4.31	0.0151	0.005		Triangle scattering
Zn-C-C	16	4.43	4.36	0.048	0.06		Triangle scattering
Zn-C	4	4.96	4.9	0.0037	0.05		Single-scattering
Zn-C-C	8	4.97	4.9	0.048	0.06		Forward-scattering

4 XPS

Table S3. XPS fitting parameter for the N1s spectra.

**Zn-N-C** Peak	Binding Energy /eV	L-G Mixing	FWHM / eV	Relative Area / %
N 1	398.3	0.2	1.6	71.1
N2	400.5	0.2	1.6	23.2
N3	402.3	0.2	1.7	5.7
**NDC** Peak	Binding Energy /eV	L-G Mixing	FWHM / eV	Relative Area / %
N 1	398.0	0.2	1.8	56,9
N2	400.2	0.2	1.8	37.0
N3	402.3	0.2	1.7	6.1

5 P-XRD

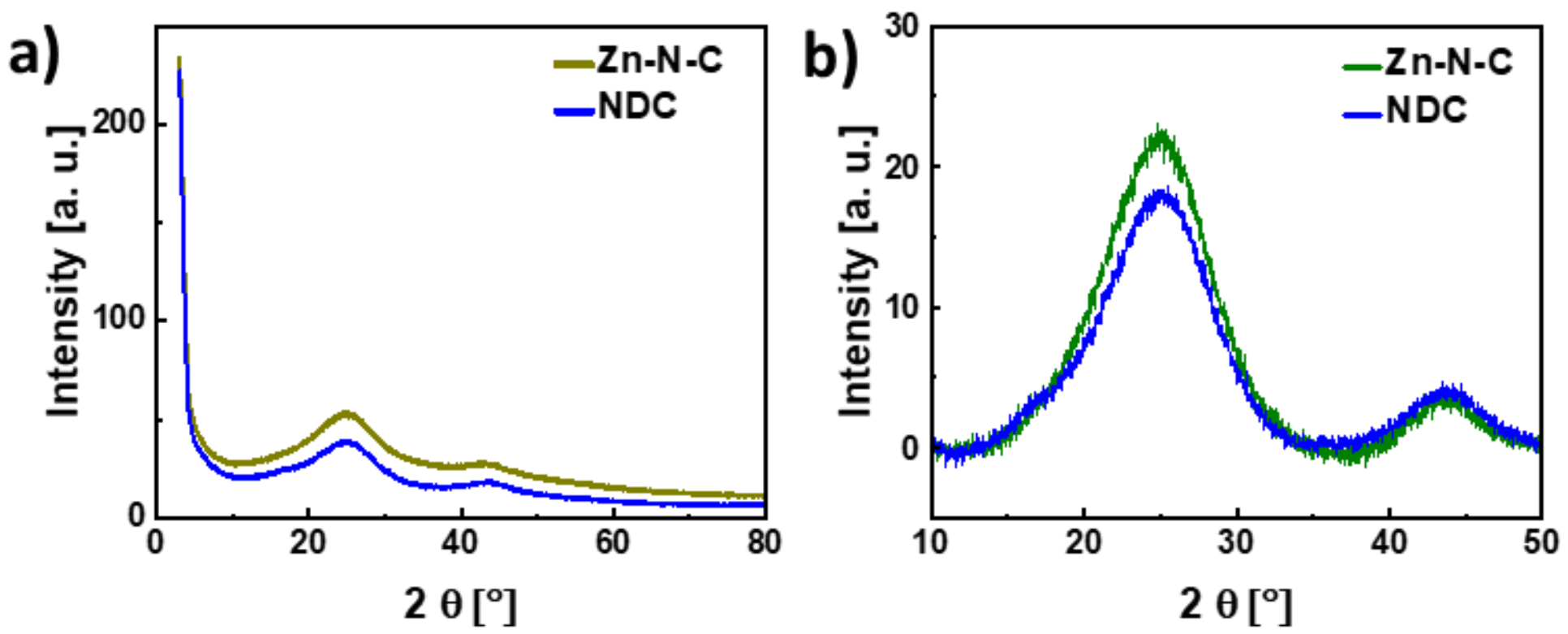

Figure S3. a) P-XRD-of Zn-N-C and NDC and b) background corrected diffractogram of Zn-N-C and NDC.

6 Gas-sorption data

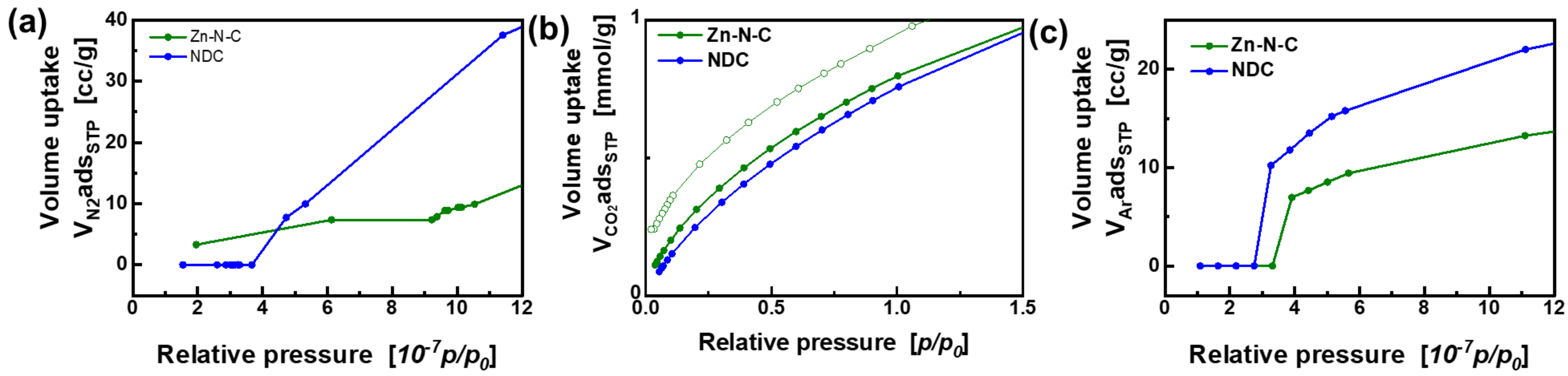

Figure S4. Initial gas-uptakes of Zn-N-C and NDC in a) N_2-isotherm, b) CO_2-isotherm and c) Ar-isotherm.

Table S4. Raw-experimental data of the N_2, CO_2 and Ar isotherms of Zn-N-C and NDC.

Zn-N-C-N_2 sorption		NDC-N_2 sorption		Zn-N-C-CO_2 sorption		NDC-CO_2 sorption		Zn-N-C-Ar sorption		NDC-Ar sorption	
p/p0	V_{N2} ads_{STP} [cc/g]	p/p0	V_{N2} ads_{STP} [cc/g]	p/p0	V_{CO2} ads_{STP} [mmol/g]	p/p0	V_{CO2} ads_{STP} [mmol/g]	p/p0	V_{Ar} ads_{STP} [cc/g]	p/p0	V_{Ar} ads_{STP} [cc/g]
1.96E-07	3.2976	1.55E-07	0	3.67E-05	0.110358	5.35E-05	0.0866785	1.09E-07	0	1.09E-07	0
6.13E-07	7.3572	2.60E-07	0	4.16E-05	0.117262	6.27E-05	0.099049	1.64E-07	0	1.64E-07	0
9.22E-07	7.3649	2.87E-07	0	4.54E-05	0.123941	6.88E-05	0.107012	2.19E-07	0	2.19E-07	0
9.37E-07	7.8725	3.03E-07	0	5.70E-05	0.141993	8.60E-05	0.129052	2.75E-07	0	2.75E-07	0
9.62E-07	8.8851	3.13E-07	0	7.16E-05	0.163622	0.0001041	0.151263	3.31E-07	0	3.27E-07	10.2249
9.72E-07	8.8864	3.24E-07	0	9.93E-05	0.200199	0.00019557	0.246581	3.91E-07	6.9642	3.85E-07	11.7802
1.00E-06	9.3929	3.30E-07	0	0.00013549	0.244895	0.00030234	0.338311	4.42E-07	7.6659	4.45E-07	13.4988
1.01E-06	9.3967	3.67E-07	0	0.00020186	0.312252	0.00038951	0.404624	5.01E-07	8.5273	5.14E-07	15.1984
1.05E-06	9.9031	4.73E-07	7.7472	0.00029255	0.389297	0.00049416	0.476074	5.66E-07	9.4257	5.56E-07	15.7775
1.82E-06	26.0598	5.32E-07	9.9674	0.00039038	0.463122	0.00059781	0.540868	1.11E-06	13.233	1.11E-06	22.0053
2.41E-06	41.2891	1.14E-06	37.5266	0.00049378	0.532551	0.00070119	0.600954	1.86E-06	16.7668	1.92E-06	27.4353
3.80E-06	60.6761	1.89E-06	54.6475	0.00059754	0.594843	0.00080421	0.656412	2.49E-06	18.3219	2.49E-06	29.9281
5.40E-06	70.7289	2.42E-06	65.4565	0.00069756	0.649886	0.00090274	0.707396	4.10E-06	22.1595	3.94E-06	35.6047
6.76E-06	74.4797	3.76E-06	82.44	0.00079766	0.701401	0.0010062	0.757719	4.13E-06	22.1999	3.98E-06	35.8001
1.35E-05	82.4687	3.90E-06	84.2259	0.00089925	0.751062	0.00191625	1.11849	5.78E-06	24.9689	5.66E-06	40.2646
3.29E-05	95.1533	5.28E-06	93.2887	0.00100274	0.797572	0.00300232	1.44514	7.12E-06	27.1955	6.88E-06	42.9405
5.22E-05	101.7856	6.81E-06	97.0238	0.00186241	1.10122	0.00394578	1.68096	1.50E-05	33.3239	1.50E-05	54.1223
7.26E-05	106.2328	1.37E-05	111.3525	0.00295029	1.38826	0.00498821	1.90648	2.53E-05	38.4388	2.46E-05	62.408
9.37E-05	109.5112	3.40E-05	131.8267	0.00390332	1.59026	0.00599744	2.10193	3.51E-05	42.1548	3.46E-05	68.6688
2.01E-04	119.1071	5.30E-05	142.1625	0.00493724	1.77717	0.00701061	2.27885	4.48E-05	45.2029	4.48E-05	73.7613
4.09E-04	127.2859	7.34E-05	149.3971	0.00596762	1.93974	0.00800951	2.43892	5.58E-05	48.1308	5.42E-05	77.7792
6.15E-04	131.6831	9.36E-05	154.775	0.00696364	2.08398	0.00901081	2.58866	6.50E-05	50.4659	6.47E-05	81.7033

8.17E-04	134.7691	1.97E-04	169.9672	0.00799337	2.21703	0.0100149	2.72867	7.45E-05	52.4572	7.51E-05	85.1772
0.00103	137.231	4.31E-04	184.5412	0.00897625	2.33479	0.0111195	2.87238	8.48E-05	54.4273	8.46E-05	88.0323
0.00204	143.9124	6.14E-04	190.7379	0.00998444	2.44793	0.0122252	3.00932	9.52E-05	56.2005	9.58E-05	91.0744
0.00403	151.2822	8.17E-04	195.415	0.0109924	2.55247	0.0133426	3.13895	2.07E-04	68.1286	1.99E-04	110.2445
0.00619	155.9477	0.00102	198.9167	0.0119925	2.6481	0.0144502	3.2602	3.05E-04	75.2626	3.06E-04	123.0117
0.008	158.6583	0.00202	209.3536	0.0129775	2.73983	0.0155561	3.37693	4.05E-04	80.1774	4.11E-04	131.6206
0.01012	161.2728	0.00409	220.3502	0.0139931	2.82968	0.0166786	3.48687	5.05E-04	84.7029	5.03E-04	137.5417
0.01612	166.8305	0.00606	226.7606	0.0149926	2.91291	0.0177743	3.59156	6.08E-04	87.85	6.04E-04	142.7529
0.02891	174.4331	0.00803	231.5688	0.0159946	2.99071	0.0188889	3.69556	7.02E-04	90.6362	6.99E-04	146.8065
0.04963	182.0684	0.01001	235.5011	0.0169831	3.06554	0.0200036	3.79508	8.12E-04	92.9773	7.99E-04	150.4783
0.07231	187.5363	0.01602	244.1352	0.0179988	3.13786	0.0211137	3.8893	8.99E-04	94.8827	9.29E-04	154.4265
0.09987	192.1734	0.03147	258.4227	0.0189875	3.20605	0.0222168	3.98153	0.00101	96.6672	0.00102	156.6909
0.14696	197.4677	0.04727	267.869	0.0199929	3.27272	0.0233372	4.07049	0.00205	106.6018	0.00203	174.1167
0.20115	201.5159	0.07836	280.074	0.0209977	3.33529	0.0244418	4.15524	0.00407	116.3957	0.00409	190.504
0.24731	204.0381	0.10032	286.1382	0.0219835	3.39626	0.0255483	4.23924	0.006	122.3878	0.00611	199.571
0.29857	206.2223	0.14705	295.4915	0.0230043	3.45465	0.026668	4.31991	0.00801	126.4846	0.00804	205.7941
0.40351	209.2837	0.19881	302.8949	0.0239747	3.5118	0.027772	4.39724	0.01018	129.9433	0.01012	210.9501
0.49873	211.4767	0.25037	308.5828	0.0250058	3.568	0.0288879	4.47168	0.01615	135.7202	0.01618	222.4357
0.59801	213.716	0.3015	313.2381	0.0259863	3.62006	0.0299786	4.54796	0.03031	144.3948	0.02971	237.7187
0.69728	216.2164	0.39852	320.7849	0.0269932	3.67183			0.0507	152.6154	0.05018	252.2886
0.80142	219.8606	0.49888	327.9732	0.0279913	3.72187			0.07464	159.1122	0.07612	264.4443
0.89928	226.7203	0.5991	334.9156	0.0290059	3.76818			0.10094	164.4065	0.10073	272.4402
0.95099	236.4943	0.69864	341.9943	0.029969	3.8157			0.14957	170.7685	0.14918	282.8055
0.99281	287.3379	0.79784	350.036	0.029969	3.8157			0.20067	175.3312	0.19911	289.583
0.99106	287.3625	0.9024	362.4336	0.0286396	3.7616			0.25002	178.3696	0.25049	294.2632
0.94955	241.943	0.95224	375.8058	0.027602	3.7174			0.30079	180.6961	0.29979	297.4642
0.89982	232.5235	0.99279	446.1883	0.0266016	3.67299			0.3996	183.5497	0.40063	301.8764
0.84932	228.633	0.9922	446.1185	0.026022	3.64637			0.50003	185.812	0.50116	305.2745
0.80052	226.1436	0.94976	379.1596	0.0250403	3.59937			0.59998	188.0346	0.60108	308.4454
0.75139	224.3806	0.89983	366.0807	0.0240341	3.55082			0.69989	190.3465	0.70086	311.7526
0.70029	222.9238	0.85095	359.2718	0.0230502	3.49945			0.80078	193.6321	0.79986	316.1363
0.64964	221.6755	0.79969	354.008	0.0220377	3.44466			0.90047	199.8471	0.90114	324.7787
0.59881	220.555	0.74991	349.6583	0.0210421	3.38921			0.95058	208.3062	0.94937	335.5241
0.54853	219.5175	0.69924	345.8002	0.0200448	3.33152			0.99411	253.6319	0.99413	439.0921
0.49936	218.3711	0.64862	342.0489	0.0190491	3.27038			0.99732	279.5933	0.99715	601.2709
0.4484	215.3264	0.5984	338.7714	0.0180218	3.21032			0.99554	277.6972	0.99586	575.2472
0.39933	214.0645	0.54862	335.3597	0.0170725	3.14702			0.94982	213.3469	0.94995	342.8664

0.34967	212.6919	0.49841	331.8745	0.0160317	3.07536			0.9003	204.8443	0.90084	329.3104
0.29998	211.1587	0.45077	326.1474	0.0150552	3.00301			0.85073	201.1184	0.85015	323.7418
0.25095	209.3194	0.39908	321.9836	0.0140476	2.92455			0.79936	198.7839	0.80049	320.4461
		0.34894	317.9663	0.0130523	2.84286			0.75097	197.2023	0.74957	317.951
		0.2991	313.8811	0.0120536	2.75611			0.7008	195.9389	0.69893	315.9938
		0.24924	309.4679	0.011052	2.66468			0.6504	194.9112	0.64871	314.302
				0.0100607	2.56727			0.60029	193.9701	0.60089	312.8388
				0.00905953	2.46123			0.55029	193.1047	0.551	311.3763
				0.0080554	2.34815			0.50018	192.2635	0.49866	309.8824
				0.00707088	2.22599			0.45052	191.1467	0.449	307.8929
				0.00606651	2.08831			0.39984	188.4025	0.39892	303.7665
				0.00506608	1.93683			0.35055	187.1408	0.34934	301.4203
				0.00407442	1.76697			0.30084	185.5806	0.29952	298.8378
				0.00309429	1.56588			0.24904	183.4896	0.25026	295.6983
				0.00204439	1.308						
				0.00105878	0.978111						
				0.00089101	0.896222						
				0.00077554	0.840809						
				0.00070983	0.806481						
				0.0006067	0.751707						
				0.00052202	0.702466						
				0.00040833	0.627868						
				0.00032153	0.564174						
				0.00021431	0.47635						
				0.00010823	0.363207						
				9.64E-05	0.346434						
				8.54E-05	0.328932						
				7.55E-05	0.313441						
				6.54E-05	0.297514						
				5.43E-05	0.278789						
				4.34E-05	0.260453						
				3.29E-05	0.241021						
				2.15E-05	0.239986						

7 Models and Results of DFT calculations:

Ar, N_2 and CO_2 adsorption:

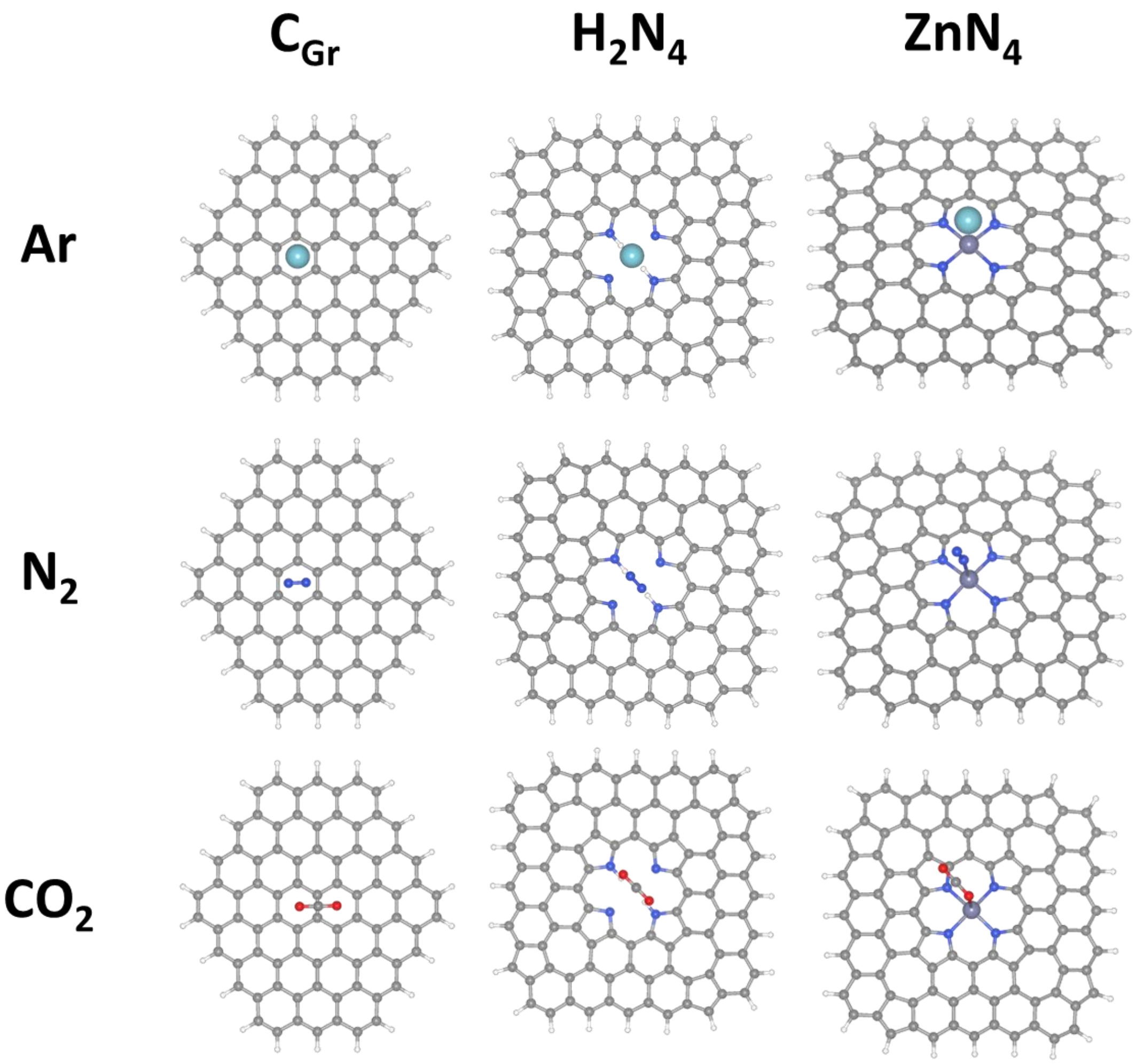

Figure S5. Adsorption geometries of Ar (top), N_2 (middle) and CO_2 (bottom) on graphitic carbon cluster C_{Gr} (left), H_2N_4 site (middle) and ZnN_4 site (right) optimized with M06-2X functional, def2-SVP basis and D3(BJ) dispersion correction.

8 Residual CO_2 adsorption quantities

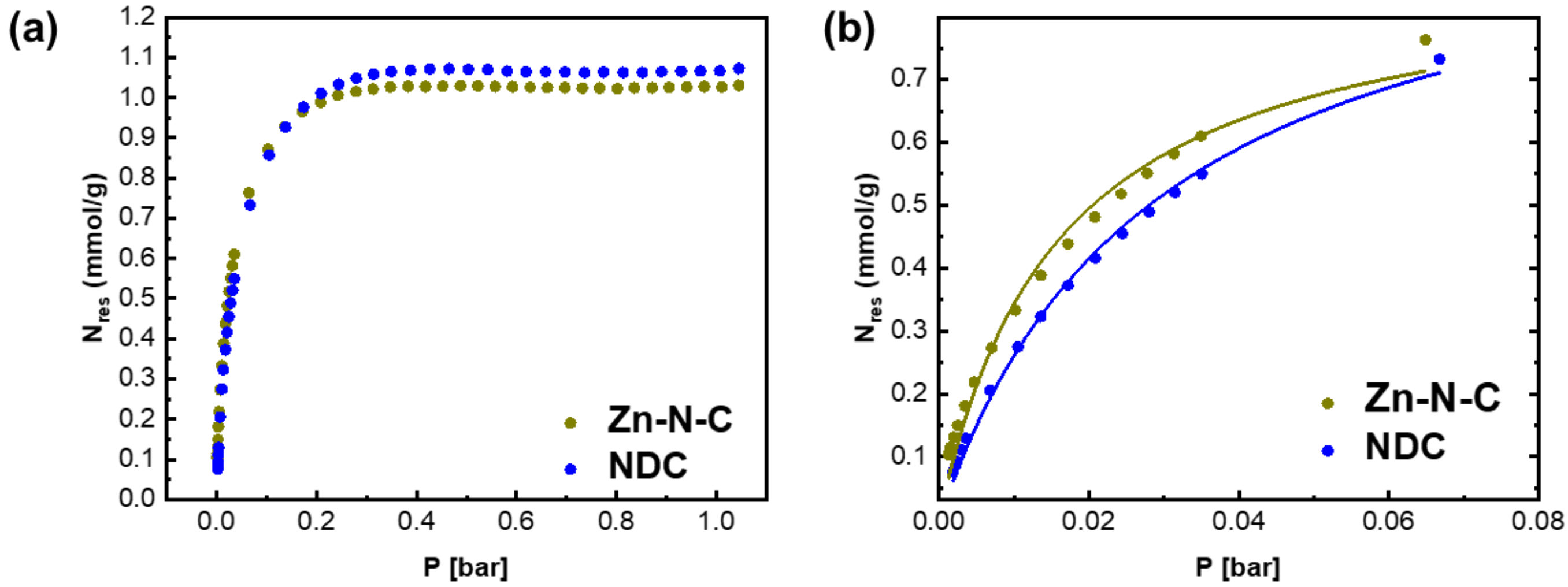

Figure S6. (a) Plot of residual CO_2 adsorption quantities obtained upon subtraction of the theoretical isotherm of the main (weak) adsorption from the raw isotherm for (a) the entire measured pressure range and (b) at low pressures ($P < 0.08$ bar). The line in (b) represents the best-fit to a single-site Langmuir equation.

9 N_2-PSD

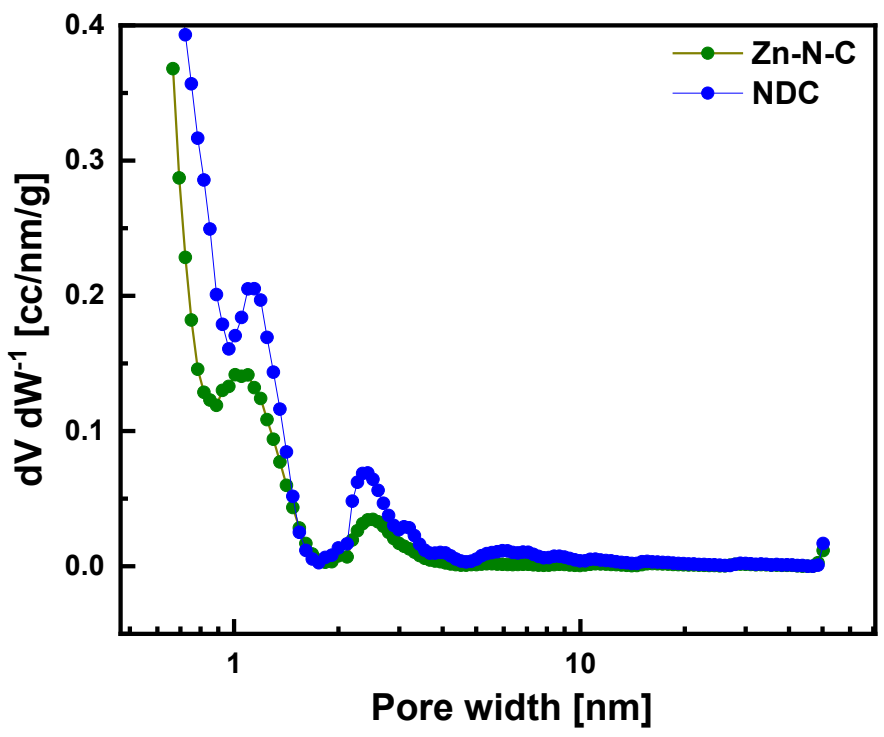

Figure S7. PSD derived starting at $p/p_0=10^{-5}$ for Zn-N-C and NDC.

10 CHNO/O-Analysis

Table S5. Elemental composition of the Zn-N-C and NDC samples. The CHN content is obtained from elemental combustion analysis and O from elemental pyrolysis analysis.

	C wt.%	H wt.%	N wt.%	O wt.%
Zn-N-C	62.05	0.64	13.94	7.81
NDC	72.49	0.82	13.46	3.48

11 Structural and Electronic analysis of adsorbed clusters

In order to elucidate the influence of quadrupole moment on the adsorption properties at respective active sites, we analyzed the binding geometry and charge distributions within the probe gas. The visual representations of the adsorbed structures are shown in Figure S5. On graphene and H_2N_4 site, both CO_2 and N_2 are adsorbed parallel to the surface in order to maximize dispersive interactions. However, ZnN_4 site shows a deviating end-on adsorption geometry that is consistent with bond angles that are expected from valence shell electron pair repulsion (VSEPR), indicating a dative-bonding character that involves the lone pair of the O and N atom in CO_2 and N_2. The C=O bond length in CO_2@ZnN_4 is also slightly asymmetrized in contrast to graphene and H_2N_4 sites. The asymmetry is further reflected in the difference in NBO charge between O_{Zn} and $O_{terminal}$, as the presence of positively charged Zn^{2+} polarizes the CO_2 electron distribution towards Zn, further supporting a dative bond character. This effect is analogously observed in the binding of N_2@ZnN_4, albeit to a smaller extent due to smaller quadrupole moment of N_2. For H_2N_4, although the adsorption of CO_2 does not lead to significant differences in C=O bond length, there is still a slight charge redistribution leading to slightly positive and negative partial charges at C and O respectively compared to free CO_2 or graphene. As the optimized CO_2 geometry is aligned parallel to the N-H bond with O atoms in close proximity with the H atoms, the slight increase in quadrupole moment within the CO_2 likely serves to enhance the interaction with the N-H dipole, although the effect is still less pronounced than on ZnN_4.

Table S6. Analysis of adsorption geometry (bond length, angles) and NBO charge distribution of the gas-adsorbed graphitic cluster C_{Gr}, H_2N_4 site and ZnN_4 site. Geometry optimization was performed with M06-2x-D3(BJ)/def2-SVP and NBO population analysis was performed on the optimized structure using M06-2x-D3(BJ)/def2-TZVP.

	**Geometry**	**C=O bond length**	**C charge**	**O charge**
Free CO_2		1.156	1.026	-0.513
CO_2@Gr	Side-on	1.157	1.039	-0.517
CO_2@H_2N_4	Side-on, parallel to N-H	1.157	1.049	-0.524
CO_2@ZnN_4	∠Zn-O-C = 115 °	1.164, 1.151	1.065	-0.570 (O_{Zn})<br>-0.482 ($O_{terminal}$)
	**Geometry**	**N=N bond length**	**N charge**	**N charge**
Free N_2		1.093	0	0
N_2@Gr		1.094	0.003	0.003
N_2@H_2N_4	Side-on parallel to N-H	1.094	-0.002	0.005
N_2@ZnN_4	∠Zn-N-N = 166 °	1.093	-0.037 (N_{Zn})	0.058 (N_{far})
			**Ar Charge**	
Ar@Gr			0.005	
Ar@H_2N_4			0.007	
Ar@ZnN_4			0.020	

Table S7. CO_2 adsorption energies on C_{Gr}, H_2N_4 site and ZnN_4 clusters as used in this work and a smaller model (denoted "small") for comparison of the cluster size effect. The size effect is calculated as the absolute difference between the CO_2 adsorption energy on the smaller model and the larger model. All energies are calculated with M06-2X-D3(BJ)/def2-TZVP without ZPE-, thermal- and counterpoise correction.

	**Chemical Formula**	**$\Delta E_{ad, CO2}$ (eV)**	**Size effect (eV)**
C_{Gr} (small)	$C_{42}H_{16}$	-0.163	0.002
C_{Gr}	$C_{80}H_{22}$	-0.165	
H_2N_4 (small)	$H_2N_4C_{52}H_{20}$	-0.239	0.009
H_2N_4	$H_2N_4C_{96}H_{24}$	-0.248	
ZnN_4 (small)	$ZnN_4C_{52}H_{20}$	-0.262	0.004
ZnN_4	$ZnN_4C_{96}H_{24}$	-0.266	

11 Supporting References

[1] J. S. Bates, F. Khamespanah, D. A. Cullen, A. A. Al-Omari, M. N. Hopkins, J. J. Martinez, T. W. Root, S. S. Stahl, *J. Am. Chem. Soc.* **2022**, *144*, 18797-18802.
[2] G. Polzonetti, A. Ferri, M. V. Russo, G. Iucci, S. Licoccia, R. Paolesse, *Journal of Vacuum Science & Technology A* **1999**, *17*, 832-839.
[3] B. Ravel, M. Newville, *Journal of Synchrotron Radiation* **2005**, *12*, 537-541.
[4] R. Ahlrichs, M. Bar, M. Haser, H. Horn, C. Kolmel, *Chem. Phys. Lett.* **1989**, *162*, 165-169.
[5] Y. Zhao, D. G. Truhlar, *Theor. Chem. Acc.* **2008**, *120*, 215-241.
[6] F. Weigend, R. Ahlrichs, *Phys. Chem. Chem. Phys.* **2005**, *7*, 3297-3305.
[7] S. F. Boys, F. Bernardi, *Mol. Phys.* **2006**, *19*, 553-566.
[8] J. L. Low, B. Paulus, *Catalysts* **2023**, *13*, 566.
[9] K. Momma, F. Izumi, *J Appl Crystallogr* **2011**, *44*, 1272-1276.
